# Measured and self-reported olfactory function in voluntary Norwegian adults

**DOI:** 10.1007/s00405-022-07298-7

**Published:** 2022-02-23

**Authors:** Ingrid Torvik Heian, Anne-Sofie Helvik, Thomas Hummel, Marte Rystad Øie, Ståle Nordgård, Mette Bratt, Wenche Moe Thorstensen

**Affiliations:** 1 Institute of Neuromedicine and Movement Science (INB), Norwegian University of Science and Technology (NTNU), Trondheim, Norway; 2 Department of Otolaryngology, Head and Neck Surgery, Molde Sjukehus, Helse Møre og Romsdal, 6412 Molde, Norway; 3 Department of Public Health and Nursing, Faculty of Medicine and Health Sciences, Norwegian University of Science and Technology (NTNU), Trondheim, Norway; 4 Norwegian National Advisory Unit on Ageing and Health, Vestfold Health Trust, Tønsberg, Norway; 5 Department of Otolaryngology Smell and Taste Clinic, TU Dresden, Dresden, Germany; 6 Department of Otolaryngology, Head and Neck Surgery, St. Olavs University Hospital, Trondheim, Norway

**Keywords:** Olfaction disorders, Prevalence, Self-report, Smell, Sniffin’ Sticks

## Abstract

**Purpose:**

The lack of epidemiological data on the proportion of olfactory dysfunction (OD) using comprehensive olfactory assessment in healthy adults in Scandinavia motivated to the present study which aimed to explore the proportion of OD in voluntary healthy Norwegian adults, assessed by Sniffin’ Sticks, and its correlation to self-reported olfactory function. Furthermore, sociodemographic and clinical factors associated with olfactory function were analysed.

**Methods:**

The sample included 405 Norwegian participants, aged 18–78 years, 273 women and 132 men, who underwent olfactory testing with extensive Sniffin’ Sticks test, allergy testing, clinical examination with nasal endoscopy and completed a self-administered questionnaire, including self-evaluation of olfactory function on a 100 mm Visual Analogue Scale.

**Results:**

We found that 37% had OD, of which 1.2% had anosmia assessed with extensive Sniffin’ Sticks test. The proportion of hyposmia and anosmia increased with age. Men and participants with low education had poorer olfactory function scores. Allergy, smoking status, general health and endoscopic findings were not associated with measured olfactory function. We found no correlation between self-reported and measured olfactory function.

**Conclusions:**

This study has identified that a large proportion of our sample of voluntary healthy Norwegian adults have OD, considerably more common in older adults and somewhat more common in men and individuals with low education. The lack of correlation between self-reported and measured olfactory function highlights the importance of using validated tests for a reliable olfactory evaluation.

## Introduction

Olfaction plays an essential role in social interaction, emotional experience, nutrition, neurological health and the ability to avoid danger. Consequently, olfactory dysfunction (OD) can contribute to social isolation, depression [1], malnutrition [2], decreased quality of life [3] and increased risk of injury [4]. Additionally, there is mounting evidence that olfaction is associated with major health outcomes, including increased risk for neurodegenerative diseases and mortality [5]. Despite this, OD remains underrecognized, underdiagnosed and undertreated in the general population [6, 7].

Several studies have shown a variable prevalence of OD, depending on sample demographics and olfactory assessment [7–10]. Apart from aging, the most common identified causes of OD are viral infection of the upper respiratory tract, sinonasal disease, traumatic head injury, neurological disease, chronic exposure to toxins and congenital OD [6, 11, 12]. However, many lack a history of typical underlying conditions, indicating that other etiological factors may play an important role in the development of this ailment. Some studies suggest that up to 16% of cases with OD are classified as idiopathic [13]. Current smoking, but not former smoking, has been associated with an increased risk of OD [14], but some studies found no such association [11]. In allergic subjects, the presence of OD seems to increase with disease severity and duration [15]. Further, male sex [16], low education level [8, 17], poor general health and cognitive impairment [17, 18] are found to be associated with OD. OD has been estimated to affect over 40% of the general aging population [7, 9, 18, 19], with the most pronounced decrease in function for people aged 61–70 years [19]. Many studies have demonstrated that women outperform men in olfactory function, but studies involving large samples have found that sex-related differences seem to be small [16, 19, 20].

The assessment of olfaction range from self-evaluation to comprehensive psychophysical tests [6]*.* The reliability of self-evaluated olfactory function is debatable [21–24] and the use of validated olfactory tests is preferential. A recent meta-analysis found that the prevalence of OD was significantly greater when olfaction was measured using psychophysical tests (28.8%), compared to subjective assessment (9.5%), and that extended olfactory test was more sensitive than brief test [7]. A comprehensive and well-established tool for olfactory assessment is the Sniffin’ Sticks test, which is validated in numerous countries worldwide [19, 25] and used in clinical practice and scientific research. It contains three subtests on threshold (T), discrimination (D) and identification (I) that give a more comprehensive understanding of olfactory function than a simpler test only assessing olfactory identification.

In a Scandinavian context, a prevalence of OD of 19.1% was found in a large study from Sweden [9] and 28% in a relatively small study of cognitively healthy middle-aged and older adults in Norway [26]. Both these studies used the Scandinavian Odor Identification Test for olfactory assessment. To the best of our knowledge, an epidemiological study using a more comprehensive assessment of olfactory function in healthy adults is lacking in Scandinavia. Moreover, epidemiological data on the proportion of OD in healthy Norwegian adults may prove important for understanding the burden of OD in this population and identifying factors affecting olfactory function. Furthermore, such data may be indicative for clinicians in how to evaluate these individuals and may contribute to prevent negative health consequences of OD.

Hence, the present study aimed to explore the proportion of OD in healthy Norwegian adults, assessed by Sniffin’ Sticks, and its correlation to self-reported olfactory function. Furthermore, sociodemographic and clinical factors associated with olfactory function were studied.

## Materials and methods

### Participants

Participants in this cross-sectional study were recruited for two independent studies via public advertisement as participants in a randomized controlled trial examining olfactory training (OT) in healthy adults (*n* = 298) and as a control group in a study on rhinosinusitis in patients with chronic obstructive pulmonary disease (COPD) (*n* = 107) [27], between 2016 and 2019.

**Fig. 1:**
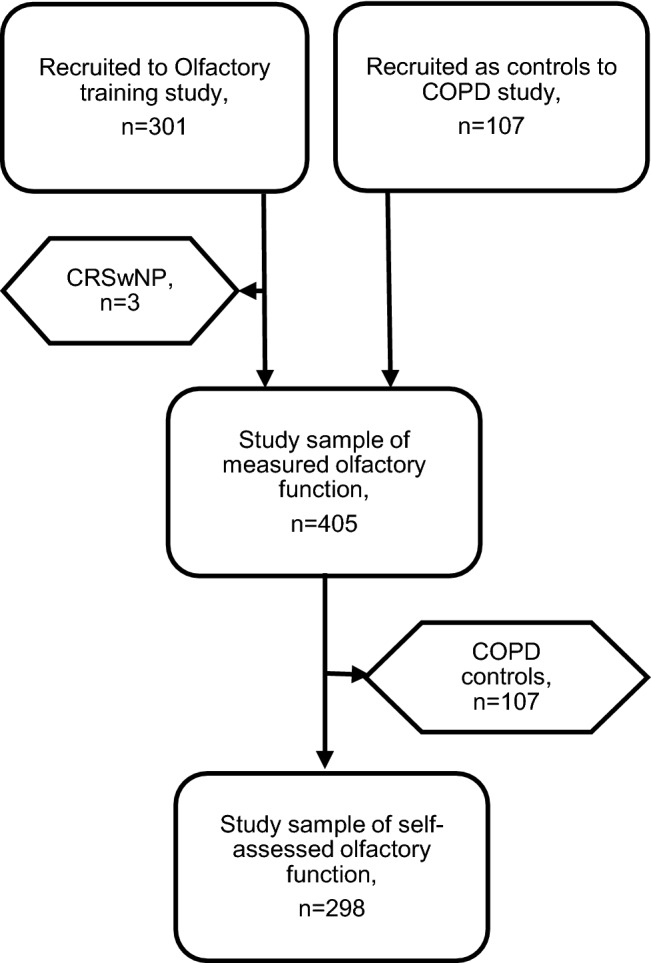
Overview of the study sample

The inclusion criteria were adults aged 18 years and above and exclusion criteria were diseases affecting olfaction, such as chronic rhinosinusitis with nasal polyps (CRSwNP), severe symptoms of allergic rhinitis, sinonasal surgery for the last 3 years prior to inclusion, recent or ongoing upper respiratory tract infection, Alzheimer’s disease, Parkinson’s disease, multiple sclerosis (MS) and COPD. In total, three participants were excluded due to CRSwNP (Fig. 1).

All participants signed an informed consent form. The study was approved by The Regional Committee for Medical Research Ethics in Mid-Norway (reference number 2016/837 and 2015/2017).

### Methods

All participants completed in the following order, a self-administered questionnaire and then underwent detailed olfactory testing, a skin prick test and a clinical examination with nasal endoscopy.

### Olfactory testing

The Sniffin’ Sticks test (Burghart Messtechnik, Wedel, Germany), a psychophysical test battery validated in numerous countries worldwide [28], was used for a detailed assessment of the olfactory function. The test consists of three subtests; T, D and I, which form the composite global olfactory score (TDI). The threshold was determined when the odorized pen (*n*-butanol) was identified among three samples with the other two pens containing the solvent propylene glycol which has little or no odor. Concentration was increased if one of the odorless pens was selected and decreased if the correct pen was identified twice in a row. The mean of the last 4 of a total of 7 reversal points was used as a detection threshold ranging from 1 to 16. In the discrimination test, three pens were presented and the participant was encouraged to discriminate one different odor from two identical odors. This was performed for 16 triplets of pens. In the identification test, the participant was presented with one single odorous pen at a time. A total of 16 pens were presented and the participant had to identify each of the odors from a list of four descriptors.

The summated TDI score from all three tests, with a maximum of 48 points (each subtest with 16 points), were used to categorize patients in terms of normosmia (score ≥ 30.75), hyposmia (score 16.25–30.5) and functional anosmia (referred to as anosmia) (score ≤ 16) [19]. Further, exclusively looking at the 16-items Sniffin’ Sticks Identification test, we defined score ≥ 12 as normosmia, a score of 9–11 as hyposmia [19] and score ≤ 8 as anosmia [29].

### Allergy status

Skin prick test with an allergy panel consisting of birch, grass and mugwort pollen, Cladosporium, house dust mite and dog, cat and horse epithelia, together with positive and negative controls was performed. A wheal diameter > 3 mm 15 min after the application was defined as a positive result [30]. Subjects with a positive test and typical symptoms of hypersensitivity on exposure to the allergen(s) were classified as allergic.

### Nasal endoscopy

Nasal endoscopy (2.7 mm, 0° True View II endoscope, Olympus, Japan), was performed by an otolaryngologist after olfactory testing, and the findings were scored using the modified Lund–Kennedy score [31] to detect the presence of edema and discharge. For statistical purposes, the findings were dichotomized to “no mucus or edema” and “presence of mucus and/or edema”.

### Self-reported olfactory function

The experience of olfactory function was assessed on a 100 mm Visual Analogue Scale (VAS), with 0 mm as “the worst possible sense of smell” and 100 mm as “the best possible sense of smell” [21]. This was applied only to the participants recruited for OT (*n* = 298).

Background variables such as age, sex, level of education (elementary school, high school, college/university) and occupation were assessed using self-report questions taken from a large epidemiological study [32]. Furthermore, general health [32] was reported using one item with four response options, and for statistical purposes, the response was dichotomized into good and not good health [33]. Information about overall satisfaction in life, one item with seven response options, ranging from extremely satisfied to extremely dissatisfied [32], was reported in the OT study sample. For statistical purposes, the response was dichotomized to satisfied (extremely satisfied to satisfied) and not satisfied (including the middle neutral response to extremely dissatisfied) [33].

### Statistical analysis

SPSS version 27 (SPSS Inc., Chicago, IL, USA) was used for statistical analysis. Continuous and categorical variables are presented as mean and standard deviation (SD) and frequencies and percentages, respectively. The assumption of normality was satisfied for all continuous variables, based on test of normality (Shapiro–Wilk), histogram and Q–Q plot. For comparative analysis regarding age and sex differences between the two included study samples, we used *t* test for independent samples and chi-square tests, respectively.

Linear regression analyses (the Enter Method) were used to explore factors associated with outcomes measures and self-reported olfactory function. Independent variables of possible interest were age, sex, allergy, smoking status, education, general health, overall satisfaction and endoscopy findings. Factors included in the adjusted linear regression analysis were those associated with the outcome with *p* < 0.15 in the unadjusted linear regression analysis. In addition, allergy and smoke variables were included as they have shown to affect olfactory performance in current literature [14, 15].

Bivariate Pearson correlation coefficients were computed to assess the correlation between self-rated and measured olfactory function.

Unless specified otherwise the alpha-level was set at 0.05.

## Results

### Sample characteristics

The overall study sample included 405 participants (273 women) with a mean (SD) age 46.5 (14.4) years (age range 18–78 years). The subjects recruited to the OT study were younger with a predominance of women compared to the subjects recruited as controls in the COPD study (*p* < 0.001). The participants’ demographics are summarized in Table 1.

**Table 1:** Characteristics of the participants

	Total sample
Total (*n*)	405
Women	273 (67.4)
Age (years)	46.5 (14.4)
Education high school	75 (18.5)
College/university	321 (79.3)
Good health^a^	377 (93.1)
Allergy^b^	97 (24.0)
Current smoking^c^	65 (16.0)
Presence of mucus/edema^d^	110 (27.2)

Data presented as *n* (%) or mean (SD)

^a^vs not good health

^b^vs no allergy

^c^vs no smoking

^d^vs no mucus/edema

### Olfactory performance

The TDI, T, D and I scores are shown in Table 2.

**Table 2:** Olfactory performance

	Total sample
TDI	31.4 (4.6)
Threshold	6.2 (2.2)
Discrimination	12.5 (2.1)
Identification	12.7 (1.9)

Data presented as mean (SD)

Using the summated TDI-score normosmia was present in 63% (*n* = 255) of the participants. OD with hyposmia was present in 35.8% (*n* = 144) and anosmia in 1.2% (*n* = 5). Categorization based on the 16-item Sniffin’ Sticks Identification test-score normosmia was present in 81% (*n* = 328), hyposmia in 15.6% (*n* = 63) and anosmia in 3.5% (*n* = 14).

**Fig. 2:**
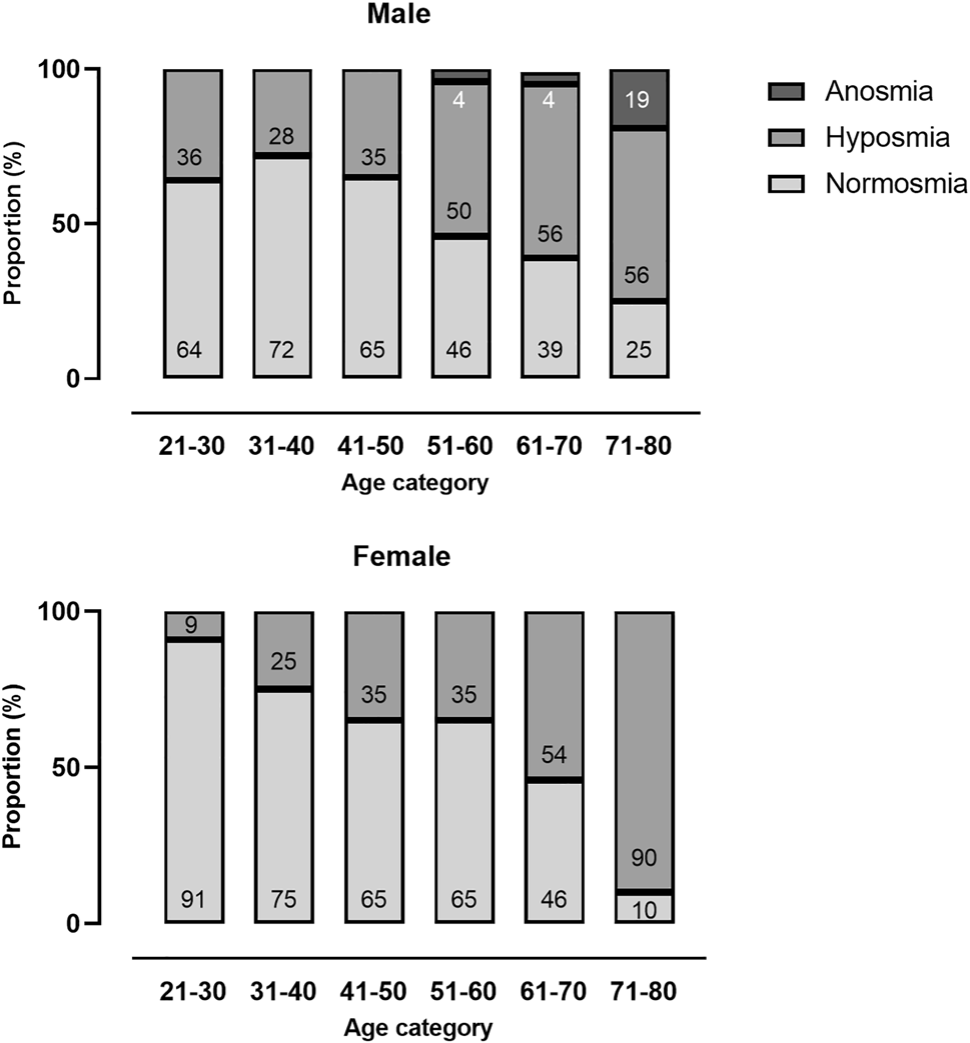
Distribution of functional anosmia, hyposmia and normosmia based on summated TDI-score, by 10-year age intervals for men and women (*n* = 405). Men: age category 21–30: *n* = 22, age category 31–40: *n* = 18, age category 41–50: *n* = 26, age category 51–60: *n* = 26, age category 61–70: *n* = 23, age category 71–80: *n* = 16. Age category 18–20: *n* = 1 is not included. Women: age category 21–30: *n* = 45, age category 31–40: *n* = 55, age category 41–50: *n* = 71, age category 51–60: *n* = 66, age category 61–70: *n* = 24, age category 71–80: *n* = 10. Age category 18–20: *n* = 2 is not included

The distribution of anosmia, hyposmia and normosmia by six age categories for men and women are presented in Fig. 2. A significant sex difference in the favor of women was present only for age category 21–30 years (*p* < 0.01).

Linear regression analysis of factors associated with the outcome variables TDI, T, D and I are presented in Table 3. In the adjusted regression analysis, there was a statistically significant association between higher age and poorer olfactory function (TDI, T, D, and I) (*p* < 0.01). Furthermore, women were more likely to have better olfactory abilities (TDI, T, D and I) than men (*p* < 0.05), and those with low education (only elementary school) were more likely to have poorer TDI, D and I compared to those with high education (college/university) (*p* < 0.05). There was no significant association between the clinical factors included (allergy, smoke, general health or endoscopic findings) and TDI, T, D and I.

**Table 3:** Unadjusted and adjusted linear regression of measured olfactory function (TDI, T, D and I)

	TDI				Threshold				Discrimination				Identification			
	*B*	95% CI		*p*	*B*	95% CI		*p*	*B*	95% CI		*p*	B	95% CI		*p*
		LL	UL			LL	UL			LL	UL			LL	UL	
**Unadjusted**																
Women^a^	2.38	1.45	3.32	< 0.01	0.85	0.41	1.29	< 0.01	0.88	0.45	1.31	< 0.01	0.58	0.18	0.97	< 0.01
Higher age, per year	− 0.14	− 0.17	− 0.11	< 0.01	− 0.05	− 0.06	− 0.04	< 0.01	− 0.05	− 0.06	− 0.04	< 0.01	− 0.04	− 0.05	− 0.03	< 0.01
Allergy^b^	0.58	− 0.48	1.63	0.29	0.12	− 0.37	0.62	0.62	0.45	− 0.03	0.93	0.07	0.04	− 0.48	0.40	0.86
Smoking^c^	− 2.28	− 3.49	− 1.07	< 0.01	− 0.72	− 1.29	− 0.16	0.01	− 0.85	− 1.40	− 0.29	< 0.01	− 0.60	− 1.11	− 0.10	0.02
Education																
Elementary school^d^	− 6.90	− 10.08	− 3.72	< 0.01	− 1.61	− 3.11	− 0.11	0.04	− 2.79	− 4.25	− 1.33	< 0.01	− 2.26	− 3.59	− 0.93	< 0.01
High-school^e^	− 1.44	− 2.59	− 0.28	0.02	− 0.60	− 1.13	− 0.06	0.03	− 0.22	− 0.75	0.31	0.42	− 0.55	− 1.03	− 0.07	0.02
Good health^f^	2.31	0.52	4.11	0.01	1.08	0.24	1.91	0.01	1.08	0.26	1.90	0.01	0.18	− 0.57	− 0.93	0.63
No mucus/edema^g^	− 1.80	− 2.80	− 0.79	< 0.01	− 0.87	− 1.33	− 0.40	< 0.01	− 0.68	− 1.14	− 0.22	< 0.01	− 0.24	− 0.66	0.18	0.26
**Adjusted**																
Women^a^	1.62	0.73	2.50	< 0.01	0.53	0.10	0.97	0.02	0.59	0.17	1.02	< 0.01	0.43	0.04	0.83	0.03
Higher age, per year	− 0.12	− 0.15	− 0.09	< 0.01	− 0.04	− 0.06	− 0.03	< 0.01	− 0.04	− 0.06	− 0.03	< 0.01	− 0.04	− 0.05	− 0.02	< 0.01
Allergy^b^	− 0.05	− 1.00	0.90	0.92	− 0.11	− 0.58	0.36	0.64	0.25	− 0.21	0.71	0.28	− 0.23	− 0.65	0.20	0.30
Smoking^c^	0.07	− 1.11	1.26	0.90	0.18	− 0.40	0.77	0.54	− 0.01	− 0.58	0.56	0.97	− 0.01	− 0.55	0.52	0.96
Education																
Elementary school^d^	− 3.68	− 6.63	− 0.72	0.02	− 0.34	− 1.80	1.12	0.65	− 1.56	− 2.99	− 0.13	0.03	− 1.55	− 2.88	− 0.23	0.02
High-school^e^	− 0.98	− 2.02	0.06	0.06	− 0.38	− 0.89	0.13	0.15	− 0.07	− 0.57	0.43	0.78	− 0.46	− 0.93	0.01	0.05
Good health^f^	1.35	− 0.31	3.00	0.11	0.86	0.05	1.680	0.04	0.71	− 0.09	1.51	0.08	− 0.14	− 0.88	0.61	0.72
No mucus/edema^g^	− 0.49	− 1.43	0.46	0.31	− 0.43	− 0.89	0.04	0.07	− 0.25	− 0.71	0.20	0.28	0.18	− 0.25	0.60	0.42

*B* Unstandardized ß coefficient for measured olfactory function; threshold (T), discrimination (D), identification (I) and total TDI

*CI* confidence interval; *LL* lower limit; *UL* upper limit. *p* *p* value

^a^vs men

^b^vs no allergy

^c^vs no smoking

^d^vs college/university

^e^vs college/university

^f^vs not good health

^g^vs mucus/edema

Total *n* = 405

### Subjective evaluation of olfactory function

The self-reported olfactory function on the VAS-score, measured for the sample recruited to the OT study, had a mean (SD) score of 69.3 (17.3). The olfactory function was rated poorer in men compared to women with a mean (SD) score of 62.4 (17.5) versus 71.5 (16.6), respectively (*p* < 0.01).

The self-reported olfactory function was not or negligibly correlated with the measured olfactory functioning scores in men and women; TDI, T, D or I [total sample: *r* = 0.009, − 0.049, 0.067 and 0.003, respectively, *p* > 0.2, men: *r* = 0.132, 0.119, 0.157 and 0.003, *p* ≥ 0.2 and women: *r* = − 0.075 (*p* = 0.3), − 0.140 (*p* = 0.04), 0.011 (*p* = 0.9) and − 0.014 (*p* = 0.8)]. Higher age, female sex, high-school educational level (vs college/university) and good health (vs not good health) were associated with a higher self-reported olfactory function in the adjusted linear regression analysis (*p* < 0.05) (Table 4). Allergy, smoking, endoscopy findings, employment and satisfaction were not associated with self-reported olfactory rating.

**Table 4:** Unadjusted and adjusted linear regression of self-reported olfactory function

Dependent variable: self-reported olfactory function (VAS 0–100)	Unadjusted				Adjusted			
	*B*	95% CI		*p*	*B*	95% CI		*p*
		LL	UL			LL	UL	
Women^a^	9.15	4.72	13.57	< 0.01	8.49	4.11	12.88	< 0.01
Higher age, per year	0.19	0.02	0.36	0.03	0.17	0.01	0.34	0.04
Allergy^b^	1.51	− 3.08	6.09	0.52				
Smoking^c^	− 3.73	− 12.00	4.54	0.38				
Education								
Elementary school^d^	− 15.44	− 39.55	8.67	0.21	− 9.56	− 33.05	13.92	0.42
High-school^e^	4.70	− 1.00	10.41	0.11	6.35	0.80	11.90	0.03
Good health^f^	10.79	1.55	20.03	0.02	9.39	0.26	18.52	0.04
Satisfied^g^	9.05	1.23	16.86	0.02	7.63	0.29	15.55	0.06
Employed^h^	2.89	− 4.22	10.00	0.43				
No mucus/edema^i^	− 1.91	− 7.03	3.21	0.46				

*B* Unstandardized ß coefficient for self-reported olfactory function

*CI* confidence interval; *LL* lower limit; *UL* upper limit. *p* *p* value

^a^vs men

^b^vs no allergy

^c^vs no smoking

^d^vs college/university

^e^vs college/university

^f^vs not good health

^g^vs not satisfied

^h^vs unemployed

^i^vs mucus/edema

Total *n* = 298

## Discussion

In the present study, we found that more than one-third of healthy voluntary Norwegian adults had OD. The proportion of hyposmia and anosmia increased with age. Men and participants with low education had poorer olfactory function scores, and allergy, smoking status, general health and endoscopic findings of edema and mucus were not associated with measured olfactory function. We found no correlation between self-reported and measured olfactory function. Women rated their olfactory function better than men, and concerning age, the findings of self-reported olfactory function were divergent compared to measured olfactory function.

In our study, 37% had OD, of which 1.2% had anosmia assessed with extensive Sniffin’ Sticks test, and 19.1% had OD, of which 3.5% had anosmia assessed with 16-items Sniffin’ Sticks Identification test. The prevalence of OD was within the range of previous studies (3.8–53%) using different assessments and age distributions [9, 10, 18, 23, 26, 34]. Even so, the prevalence in the present study is higher than in a recent meta-analysis, where the pooled prevalence of OD was estimated to be 22% in an adult healthy general population with a mean age of 63.5 years [7], and higher than in the previous published Norwegian study of middle-aged adults (28%, of which 3.4% had anosmia) [26]. However, in the meta-analysis a wide variety of studies with different olfactory assessment tools were included, and in the Norwegian study only olfactory identification was tested in a relatively small sample. More comprehensive assessments of olfactory function, like the Sniffin’ Sticks test, with all subtests (T, D and I) included, are found to estimate the prevalence of OD higher than subjective measures and more simple assessments [7]. This is supported by our study where we found a smaller proportion of participants with OD when we examined exclusively the 16-items Sniffin’ Sticks Identification test. Nevertheless, we found a larger amount with anosmia compared to when we studied the summated TDI-score. A person classified as anosmic based on identification test, may still have an olfactory function useful in daily life, being able to detect and discriminate odors, even though they cannot identify them. Detailed olfactory assessment can be valuable for detecting potential isolated deficits in subtests that may indicate the underlying disease etiology [6, 35]. When the degree of olfactory performance was assessed, only five participants rated in the lower third of the scale, and with the lack of an established cut-off for OD, we did not estimate the experienced subjective OD. However, there was no correlation between self-reported and measured olfactory performance in the present study, as found in several other studies on healthy individuals and patient populations [21, 22, 26, 36, 37]. Increased olfactory awareness in women [38] and unawareness of decline in olfactory function during aging [22, 26] may explain the higher olfactory self-rating scores presented in these groups.

In the adjusted regression analysis of psychophysically assessed olfactory function, women were more likely to have better olfactory performance for all subtests. This is in line with a meta-analysis stating that women outperform men in all aspects of olfaction, although the effect size was most prominent for olfactory threshold [16]. A meta-analysis that explored the effect of sex on olfactory identification found only a better olfactory performance in younger women aged 18–50 and explained these results with the role estrogen levels may play on olfaction [20]. The role of estrogen in olfactory function is, however, debatable [39, 40].

Our findings of a significant sex difference only in the age group 21–30 years support the idea of sex-related differences in olfactory function mostly in younger age categories. A pronounced decline in olfactory function was shown for both men and women middle-aged participants. Less than 50% of men above 50 years and women above 60 years of age were classified as normosmic (Fig. 2). The observed decline in olfactory function with increasing age found in our study, applied to all subtests (T, D and I), indicate that aging influences both peripheral and central structures of the olfactory pathway [7, 35, 41]. Another large study found age-related decline for all subtests, most pronounced for threshold [19]. This is in line with our findings.

We found significantly poorer olfactory scores in TDI, D and I, among participants with lower education compared to those with high education. The difference was not significant for T. I and D are thought to be related to higher cognitive processes, compared to T, which is primarily driven by peripheral perceptual function [41].

A strength of this study is the inclusion of healthy participants, which can better demonstrate the physiological, age-dependent decline of olfactory performance without the influence of disease-related reduction. Other strengths are the use of the validated and reliable Sniffin’ Sticks test [19] and that all participants underwent nasal endoscopy. One limitation of the study is that random sampling of the population would give a more reliable prevalence estimation than voluntary recruitment, which possibly attracts participants with particular interest in olfaction as well as the healthiest part of the population. A longitudinal study over years would be superior to assess the change of olfactory function by age. Second, we cannot rule out that some of the participants in our study had disease-related decline in olfactory function. Participants with chronic rhinosinusitis without nasal polyps may be a source of uncertainty, even if this group has shown to have olfactory function like control subject with the exception of decreased olfactory threshold [42]. Furthermore, the long-term effect of nasal surgery on OD may be debated, since this may both increase and decrease olfactory function [36]. Moreover, allergic subjects without typical symptoms of hypersensitivity on exposure may have been misclassified as non-allergic, which could have been revealed using nasal provocation test. Additionally, the Sniffin’ Sticks test’s identification subtest is not explicitly validated for the Norwegian population, introducing some insecurity of our study findings [26, 43]. Furthermore, more nuanced information could be achieved using more comprehensive questionnaires regarding olfactory-specific outcome measures, such as the Questionnaire of Olfactory Disorders (QOD) or SNOT-22 [6], as well as a validated scale for self-reported olfactory function [44] with the opportunity to categorize subjective olfactory function. Finally, information regarding co-morbidity [8], medication [10, 45] and psychological health [1] could have been included for better assessment of potential confounders.

Knowledge on the high proportion of OD in the general population, especially in the elderly, regardless of self-perceived olfactory function, may have implications for clinicians in evaluating these individuals. For the individual itself, becoming aware of the impairment may be crucial to avoid hazardous events and motivate for treatment and coping strategies to reduce the practical and social problems related to smell loss and thus improve quality of life.

## Conclusion

This study demonstrate that a large proportion of voluntary healthy Norwegian adults have OD, considerably more common in elderly adults and somewhat more common in men and individuals with low education. Furthermore, it highlights the importance of using comprehensive and validated tests for a reliable olfactory evaluation, as more simple assessment methods likely underestimate the true prevalence of OD. Appropriate evaluation and treatment of individuals with OD may reduce adverse health effects of olfactory loss, both from an individual and a public health perspective.
